# Efficient immunoaffinity chromatography of lymphocytes directly from whole blood

**DOI:** 10.1038/s41598-018-34589-z

**Published:** 2018-11-13

**Authors:** Fabian Mohr, Sabine Przibilla, Franziska Leonhardt, Christian Stemberger, Stefan Dreher, Thomas R. Müller, Simon P. Fräßle, Georg P. Schmidt, Marie-Luise Kiene, Herbert Stadler, Dirk H. Busch

**Affiliations:** 1 Institute for Medical Microbiology, Immunology, and Hygiene, Technische Universität München (TUM), Munich, Germany; 2 IBA GmbH, Göttingen, Germany; 3 National Center for Infection Research (DZIF), Munich, Germany; 4 Focus Group “Clinical Cell Processing and Purification,” Institute for Advanced Study, TUM, Munich, Germany; 5 Juno Cell Therapeutics GmbH, A Celgene Company, Munich, Germany; 6 Klinik und Poliklinik für Frauenheilkunde, Technische Universität München (TUM), Munich, Germany

## Abstract

We show that defined lymphocytes can be rapidly purified by immunoaffinity chromatography starting directly from whole blood. The method relies on low-affinity Fab-fragments attached to a column-matrix combined with the reversible Strep-tag technology. Compared to established cell enrichment protocols, the Strep-tag affinity chromatography of cells is independent of erythrocyte lysis or centrifugation steps, allowing for simple cell-enrichment with good yields, high purities, and excellent functionality of purified cells.

## Introduction

The quick and non-altering processing of cell products is an essential procedure in clinical and basic research. In the last two decades, isolation of cells from blood has been carried out using high-affinity antibodies and magnetic bead technology^1,2^. Although good purities and reasonable yields can often be obtained, major disadvantages remain, like compromising biological interference of non-reversible selection reagents (high-affinity antibodies), the need for pre-enrichment, as well as the difficulty in purifying complex multiparametric cell populations by positive selection^3^. Cell isolation protocols based on negative selection circumvent these limitations but have the drawback of often being not as ‘specific’ and ‘pure’ as compared to positive enrichment^4^. Therefore, we intended to develop a positive cell isolation method, which would overcome the major obstacles of conventional isolation methods.

We previously described a method using reversible Fab multimers, which allowed the positive enrichment of cells and subsequent release of isolation reagents from the cell surface using D-Biotin^3,5^. This approach can preserve the original functionality of purified cells *in vitro* as well as upon *in vivo* transfer and engraftment^5,6^. While the cell isolation using reversible Fab multimers could overcome some problems otherwise caused by high-affinity antibodies, whole blood specimens still required removal of erythrocytes before the selection process. This can be achieved either by density gradient centrifugation or osmotic lysis of red blood cells. Density centrifugation unfortunately often goes along with a significant loss of lymphocytes, with a reported mean recovery rate around 65%^7^, and potentially alters cell competence due to hyperosmolarity of the used reagents^8^. Direct erythrocyte lysis affects lymphocyte viability, cell composition and thereby potentially functionality^9^. Furthermore, released erythrocyte components can interfere with assay systems^10–12^. In order to improve the isolation of cells from whole blood for therapeutic, diagnostic or research applications, it would be desirable to maximize the cell yield of enrichment by minimizing initial cell loss during PBMCs generation, as well as to prevent altering the results of the subsequent analyses by skewing of functional properties of cells by antibodies. First attempts in this field have already focused on immunoaffinity chromatography (IAC)-like procedures^13–15^, but did not lead to broader applicability in basic research or clinical studies despite the potential advantages. With this report, we describe the successful transfer of a well-established platform for protein purification (Strep-tag based affinity chromatography)^16,17^ to direct processing and isolation of cells from whole blood reducing the processing times to a minimum and still providing high yields and purities.

**Figure 1:**
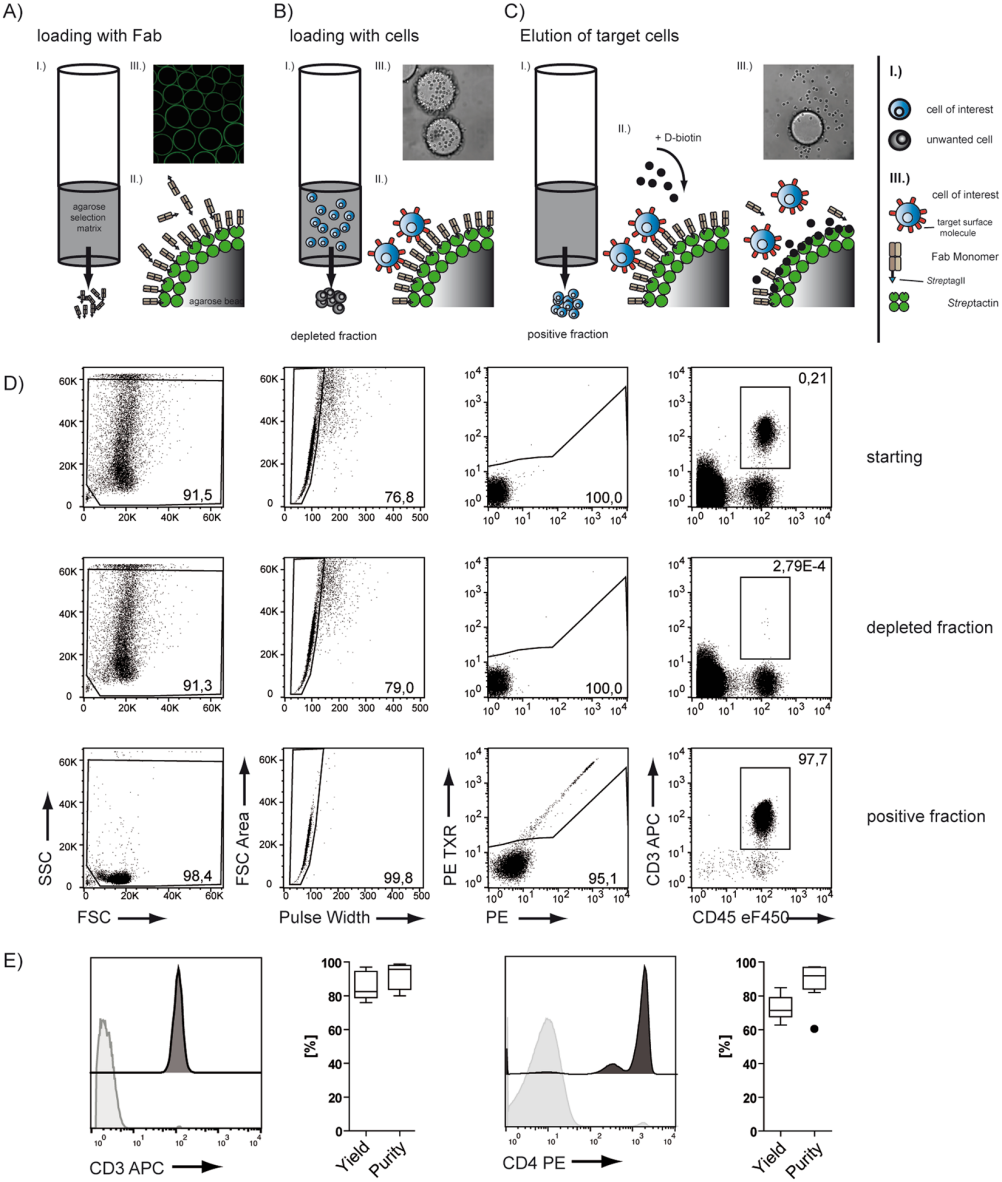
Enrichment of lymphocytes using affinity chromatography. (**A**) Schematic overview of the enrichment procedure: (I) loading of the Fab. (II) Fab molecules are coating the column matrix. (III) Strep-tagged eGFP binds to the bead surface. (**B**) (I) Single cell suspension is applied onto the column (II) specific binding of target cells, based on the Fab specificity; (III) bound cells on a bead in bright field microscopy; (**C**) (I) Biotin is flushed through the column; (II) displacing the Fab molecules on the bead surface and leading to detachment of cells; (III) detaching cells from the bead in bright field microscopy after Biotin addition. (**D**) Exemplary enrichment of CD3 positive cells, showing the gating strategy; cells are gated on single, living events excluding debris and stained for CD3. (**E**) Exemplary FACS plots depicting starting fraction (light grey) and positive fraction (dark grey) and quantification of multiple enrichments showing the yield and purity for CD3 (n = 6), and CD4 (n = 8) enrichment from whole blood. Box-and-Whisker plot: Tukey.

The Strep-tag based immunoaffinity chromatography we developed is based on an isolation matrix consisting of agarose beads, functionalized with Strep-Tactin on the bead surface (cell-grade agarose). This cell-grade agarose was filled in plastic mini-columns with frits similar to commonly used GE PD-10 desalting columns and subsequently coated with recombinant strep-tagged Fab-fragments targeting the desired cell-specific surface marker (Fig. 1A). Afterwards, a cell suspension, like whole blood, is pipetted directly onto the column and soaked into/through the column by gravity flow. At this step, marker-positive cells are held back in the column by binding to the Fab molecules on the bead surface, whereas other cells pass through. To elute remaining marker-negative cells, the column is washed with four column volumes wash buffer. (Fig. 1B). For elution of target cells, 1 mM Biotin Elution Buffer is added, which leads to rapid disruption of the Fab Strep-Tactin binding and thereby the release of cells from the matrix. Remaining monomeric Fab molecules on the target cell surface subsequently dissociate due to their low affinity and are washed away (Fig. 1C).

As a first proof-of-concept that the outlined procedure is indeed capable of realizing immunoaffinity chromatography of defined cell populations, we charged the column with anti-CD3 Fab to enrich CD3^+^ T cells from human whole blood samples. Immunoaffinity chromatography resulted in a ‘positive enrichment fraction’ of highly pure CD3^+^ T lymphocytes, whereas the ‘flow-through fraction’ was almost entirely liberated from this cell population, demonstrating a very high purification efficiency and yield (Fig. 1D). Very similar results could be obtained for multiple independent experiments and also for the enrichment of CD4-positive cells (monocytes (CD4^low^) and T cells (CD4^high^)) (Fig. 1E), showing the robustness and versatility of the method. With overall cell-processing times of usually considerably below one hour (the total processing time depends primarily on the sample volume; e.g. an enrichment of CD3-positive cells out of 10 ml whole blood/buffy coat takes 40 minutes), IAC allows enriching cells faster than currently available cell separation methods, thereby making no compromises on overall yield and purity of the enrichment process. To make sure that these encouraging results are not limited to T lymphocyte enrichment, we further broadened targeting reagents to enrich for CD19^+^ B cells, CD14^+^ cells (expressed on monocytes and granulocytes) or CD31^+^ human umbilical vein endothelial cells (HUVECs), directly from the digested vein solution. All populations were enriched with similarly high purities and yields (Fig. S1A,B,C). Next, we analyzed whether different sources of starting material (fresh whole blood, buffy coat, human cord blood, single cell suspensions from cell cultures, and splenocyte solutions from mice) could be processed with this new method. And indeed, from all tested cell specimens, we could enrich target populations with high yields and purities (Figs S1,S2,S3). Coating the affinity-matrix with streptag-ed epitope/MHC, I complexes^5,18,19^ even allow to enrich antigen-specific CD8^+^ cells with high purity and specificity (Fig. S3), demonstrating that the approach is not limited to antibody-derived Fab fragments as isolation reagents.

**Figure 2:**
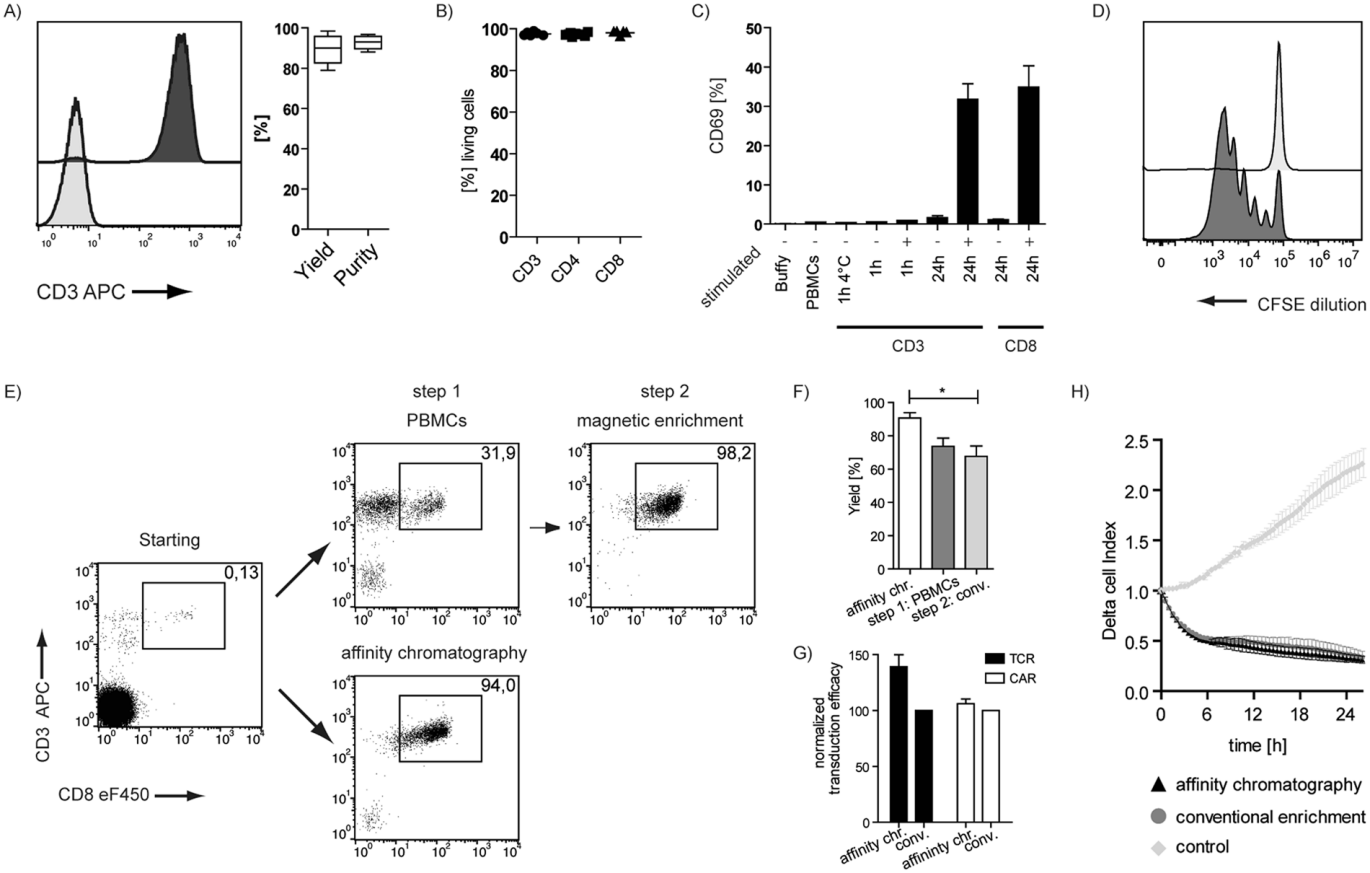
Functionality of cells after affinity chromatography. (**A**) Exemplary FACS plot depicting starting fraction (light grey) and positive fraction (dark grey) and quantification of multiple experiments showing the yield and purity for CD3 enrichment from buffy coats (n = 6). (**B)** [%] living cells after CD3, CD4 or CD8 enrichment. (**C**) CD69 expression levels upon cell selection and subsequent *in vitro* culture under different conditions; n = 3; representative of 2 experiments. (**D**) CFSE dilution at day 3 after isolation; dark grey + Expamer + IL2; light grey, only IL2. (**E**) Isolation of CD8 positive cells using affinity chromatography (1 step procedure) or conventional magnetic enrichment (2 step procedure) – exemplary FACS plots. (**F**) Yield of multiple CD8 enrichments (n = 3); One Way Anova; Tukey’s Multiple Comparison Test (**G)** Normalized transduction efficacy at day 7 using anti-CD19 CARs (n = 4; individual experiments)/TCR (n = 6; data pooled from 2 experiments). (**H)** Killing of CD19^+^ HEK target cells by anti-CD19 CAR T cells measured on the xCELLigence platform, n = 3 representative of two experiments. Box-and-Whisker plot: Tukey; Bar Graphs + SD.

To scale up the number of processed cells, we used buffy coats as starting material. For larger cell numbers IAC decreases the relative processing times per target cell even more dramatically, thereby retaining the high purity and yield of the enrichment for CD3-positive T cells (Fig. 2A). We further show that enriched cells are neither harmed nor functionally impaired by IAC (Fig. 2B–H). Therefore, we first determined the viability of purified T cells. We found that for CD3, CD4 or CD8 targeted enrichment the overall survival was in all cases very high (<97%) (Fig. 2B), indicating that there is no detectable impact on cell viability. This is also true for other cell types; even the relatively harsh collagen-based tissue mobilization procedure of HUVECs can provide good survival of primary isolated cells (Fig. S1D).

In order to analyze whether the Fab-based IAC might influence the quality of separated cells, we further explored functional parameters. As the T cell-specific Fab fragment’s parental antibody clone (CD3 clone: OKT3) is known to activate T cells, we checked for the upregulation of CD69, as an early activation marker, and the induction of proliferation by CFSE as a later activation marker of T cells after isolation of CD3 positive T cells. As shown in Fig. 2C, we could not detect any signs of CD69 upregulation on CD3^+^ target cells, analyzed 1 h and 24 h after isolation; however, controls that were re-stimulated *in vitro* by addition of the OKT3 monoclonal antibody showed a strong CD69 upregulation after 24 h. Similarly, anti-CD8 isolation also did not show any signs of CD69 upregulation through the selection procedure. In addition, no changes in CFSE expression could be observed without further *in vitro* stimulation (Fig. 2D). Overall, these data demonstrate that separation via Fab-based immunoaffinity chromatography did not lead to any detectable changes in cell viability or cell activation.

In order to test whether cell immunoaffinity chromatography is comparable to current standard technologies, we performed Fab-based IAC in parallel to a conventional, commercially available, two-step isolation process (density gradient separation of the blood samples followed by enrichment with paramagnetic beads) on the same donor samples. Both isolation processes resulted in a final product with high target cell purity. However, the conventional enrichment was characterized by significantly lower target cell yields (Fig. 2E,F), which was mainly due to the cell loss during initial PBMC generation.

After demonstrating the general effectivness of Fab-based enrichment and the ‘unchanged’ phenotype of purified cell products, we wanted to test the procedure in settings with direct clinical relevance. Adoptive T cell therapy (ACT) has proven to be a potentially curative treatment for different diseases. Antigen-specific redirection of T cells by introduction of a new T cell receptor (TCR) or chimeric antigen receptor (CAR) has the potential to improve flexibility, applicability and therapeutic outcome of ACT^20–22^. The enrichment of defined lymphocyte subsets is becoming increasingly crucial for the generation of these engineered immune cells. To asses the applicability of IAC enrichment for ACT, we compared retroviral transduction-efficacy of CD3^+^ T cells enriched by IAC to conventional methods. Transgenic expression of CMV-specific TCRs or CAR (αCD19) resulted in very similar transduction efficacies for both enrichment procedures (Fig. 2G). Also when testing the engineered cell products functionally in target cell killing assays, no significant differences depending on the enrichment procedure could be detected (Fig. 2H). These data demonstrate that cell affinity chromatography is also fully compatible with current genetic engineering approaches.

Here we report for the first time a method combining immunoaffinity chromatography and the advantages of the reversible Streptag/biotin system for cell enrichment. Cellgrade Agarose and target antigen-specific Fab/MHC-streptag fusion-proteins act as an affinity matrix to bind the cell population of interest. After the addition of low concentrations of D-biotin, the binding to the matrix is rapidly reversed and cells can be eluted. Merging the advantages of reversible cell labeling with classical IAC results in a new cell isolation technology that combines simple handling with fast processing times and can be used for cell enrichment directly from whole blood, buffy coat- or apheresis-samples, circumventing the requirement of centrifugation-based density gradient PBMC generation. This novel approach should be attractive for basic research, diagnostic and therapeutic applications and can be transferred to completely automated cell selection platforms.

## Methods

### Immunoaffinity chromatography (IAC)

Cell-grade agarose (IBA GmbH; Göttingen, Germany) was filled into plastic columns and enclosed by an upper and lower frit (GE Healthcare) in PBS. After removal of air from the column matrix and frits by application of vacuum the columns were ready to use. The frits have been de-aired using 80% Ethanol before fitting them into the columns.

The columns were coated with recombinant strep-tagged Fab-fragments/MHC-I molecules targeting the desired cell-specific surface marker for 5 minutes. Afterwards, a single cell suspension is pipetted directly onto the column and soaked into/through the column by gravity flow. The column is washed with four column volumes wash buffer (PBS/0.5% BSA/1 mM EDTA, ph 7.4). 20 ml D-biotin elution buffer (PBS/0.5% BSA/1 mM EDTA/1 mM D-biotin; ph 7.4) is added, releasing the cells from the matrix.

### Blood samples

Peripheral blood samples (whole blood sampels) were obtained from healthy adult donors of both sexes at the Institute of Medical Microbiology, Immunology and Hygiene (Technical University of Munich), and buffy-coats were obtained from autologous male or female blood donors (18–82 years old) at the Institute for Anesthesiology, German Heart Centre Munich (State of Bavaria and Technical University Munich). Cord Blood samples were obtained by the Klinik und Poliklinik für Frauenheilkunde, Technical University of Munich, Munich, Germany. The written informed consent was obtained from the donors, and usage of the blood samples was approved according to national law by the local Institutional Review Board (Ethikkommission der Medizinischen Fakultät der Technischen Universität München). To all blood specimens and single cell solutions, anti-coagulant was added during the initial processing steps. We used either Heparin (for whole blood and cord blood samples), acid-citrate-dextrose to buffy coat samples or EDTA (1 mM) to splenocyte single cell solutions.

### Fab monomer generation

The monomeric Fab Fragments were produced as described previously^3^. In short, monomeric Fab fragments originating from monoclonal antibodies were generated by gene synthesis (Invitrogen) or by PCR-based cloning of the variable region from hybridomas. The parental clones used are aCD3:OKT3^23^; aCD4:13B8.2^24^; aCD8:OKT8^23^; aCD14:2F9 (iba GmbH; Göttingen; Germany), aCD19:4G7^25^ and aCD31:10F7 (Synaptic Systems, Göttingen, Germany). Following the cloning, Fab Monomers were periplasmatically expressed in *E.coli* and the recombinant protein harvested as described before^26,27^; subsequently, Fab-fragments were purified by Strep-tag/Strep-Tactin affinity chromatography via a Strep-Tactin Superflow column (IBA GmbH, Göttingen, Germany) and stored in PBS pH 7.5^17^. After generation and verification, in some cases mutagenesis PCR was applied to introduce single amino acid substitutions within the non-hypervariable framework regions^28^ to reduce the affinity of monomeric Fabs.

### FACS analysis

For FACS analysis cells were stained for 20 minutes at 4 °C by the application of the respective antibodies: aCD4 PE (RM4-5), aCD8 PE-Cyanine7 (53-6.7), aCD3 APC (OKT3), aCD14 APC (61D3), aCD31 (WM-59) (all from eBioscience; Frankfurt am Main, Germany). For live dead discrimination propidium iodide (Molecular Probes, Eugene, Oregon) was used. MHC-I staining was performed for 45 minutes at 4 °C using Strept-Tactin APC backbone (IBA GmbH, Göttingen, Germany). Data were collected by flow cytometry on a CyAn ADP Lx (Beckman Coulter) and analyzed with FlowJo software (TreeStar). Absolute cell numbers were calculated using 123 count beads (Thermo Fisher Scientific, Waltham, USA).

### Retroviral transduction

T cells were retrovirally transduced with already published protocols^29,30^.

### xCelligence kill assay

2 × 10^4^ CD19 presenting Human embryonic kidney cells (HEK293-CD19^+^) were seated into E-Plate 96 (ACEA, Biosciences Inc. San Diego. USA). After resting CAR-positive cells were added in a 1:1 ratio and incubated for 2 days while measuring. For analysis, only the first 26 h were analyzed, as afterwards unspecific cell death was observed.

### CFSE dilution assay

Cells were isolated using CD3 IAC and stained with CFSE (Thermo Fisher Scientific, Waltham, USA) according to manufacturer’s protocol. Afterwards the cells were stimulated with CD3/CD28 Streptamers for T cell expansion (IBA GmbH, Göttingen Germany) according to manufacturer’s protocol for three days and proliferation was assessed using FACS analysis.

### Conventional cell enrichment

PBMCs were generated using Biocoll Separating Solution (Dens 1.077 g/ml) (Biochrom GmbH, Berlin, Germany) according to manufacturer’s protocol. Cells were positively enriched using the LS columns and CD8 MicroBeads according to manufacturer’s protocol (Miltenyi Biotec, Bergisch Gladbach, Germany).

## Electronic supplementary material

Supplemental Information
