# Supplementary material for: Efficient immunoaffinity chromatography of lymphocytes directly from whole blood
Source: Sci Rep. 2018 Nov 13;8:16731. doi: 10.1038/s41598-018-34589-z (PMC6233198; doi:10.1038/s41598-018-34589-z)
Supplement: Supplementary file 1 — Supplemental Information [file 41598_2018_34589_MOESM1_ESM.pdf]

Figure S1

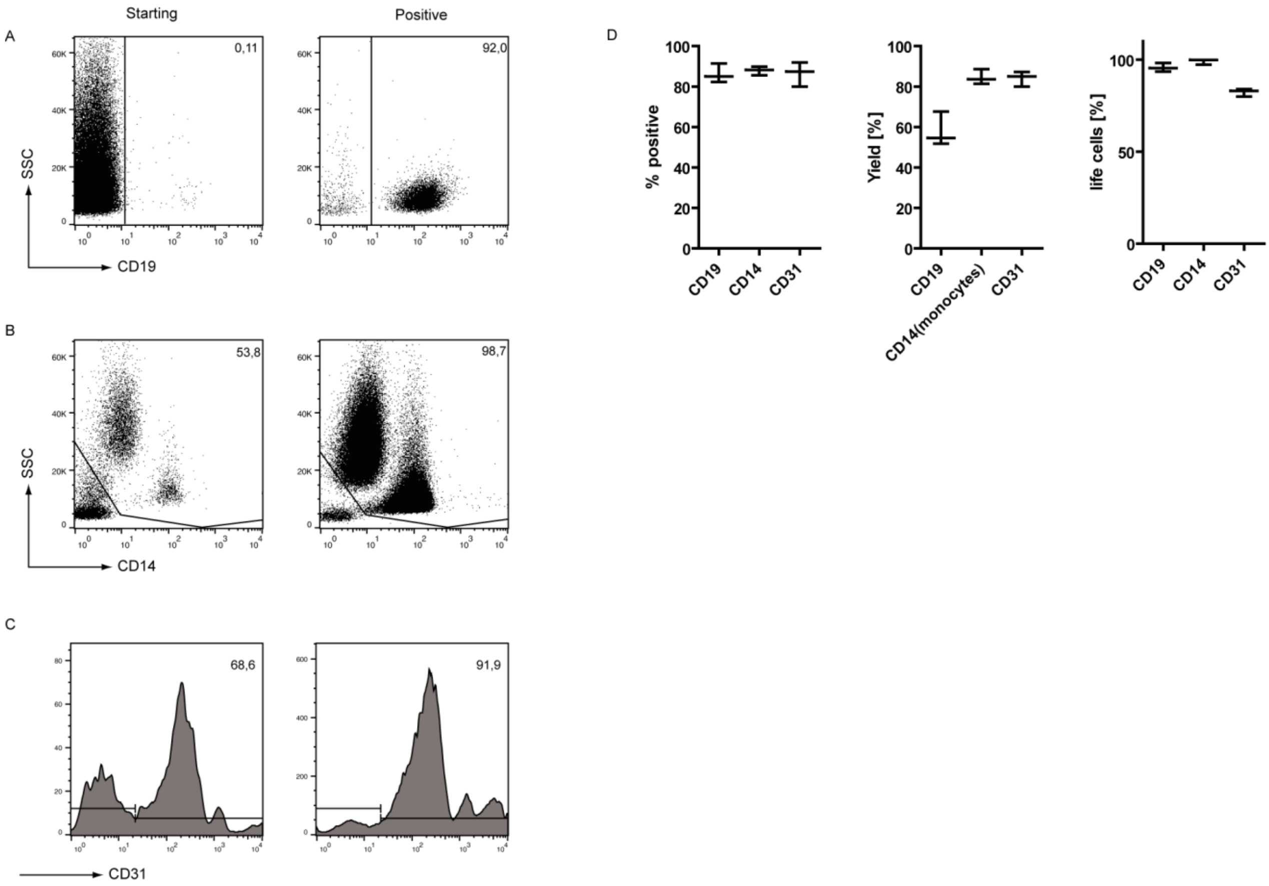

Figure S1. Enrichment of different cell populations using affinity chromatography: A) Enrichment of CD19 positive cells from whole blood; cells are gated on single, living events excluding debris and stained for CD19. **B)** Enrichment of CD14 positive cells from whole blood; cells are gated on CD45 positive, single, living events excluding debris and stained for CD14. **C)** Enrichment of CD31+ human umbilical cords vein cells from digested and washed umbilical cord veins, pre-gated on living, non-erythrocyte cells, excluding debris and stained for CD31 **D)** quantification of multiple enrichments showing the yield and purity and %living cells after the enrichment. Box-and-Whisker plot: Tukey (n=3)

Figure S2: Different Donor Material

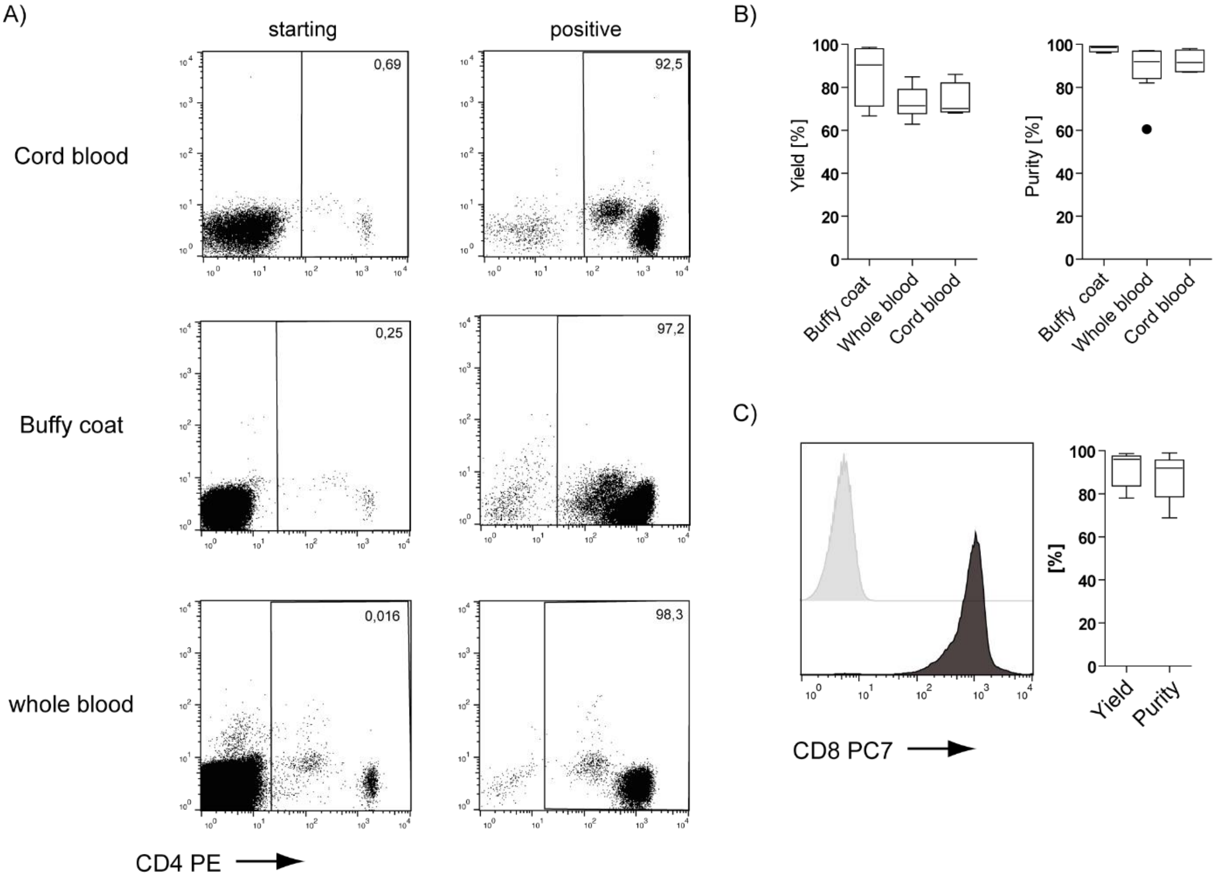

Enrichment of cell populations from different donor material using affinity chromatography: **A)**Exemplary enrichment of CD4 positive cells from different donor materials, showing the gating strategy; cells are gated on single, living events excluding debris and stained for CD4 B) quantification of multiple enrichments showing the yield and purity for CD4 IAC for enrichment from whole blood (n=8), buffy coat (n=4) and cord blood (n=4) C) Exemplary FACS blots depicting starting fraction (light grey) and positive fraction (dark grey) and quantification of multiple enrichments showing the yield and purity for CD8 enrichment from buffy coat (n=5).

Figure S3 Enrichment of MHC positive cells:

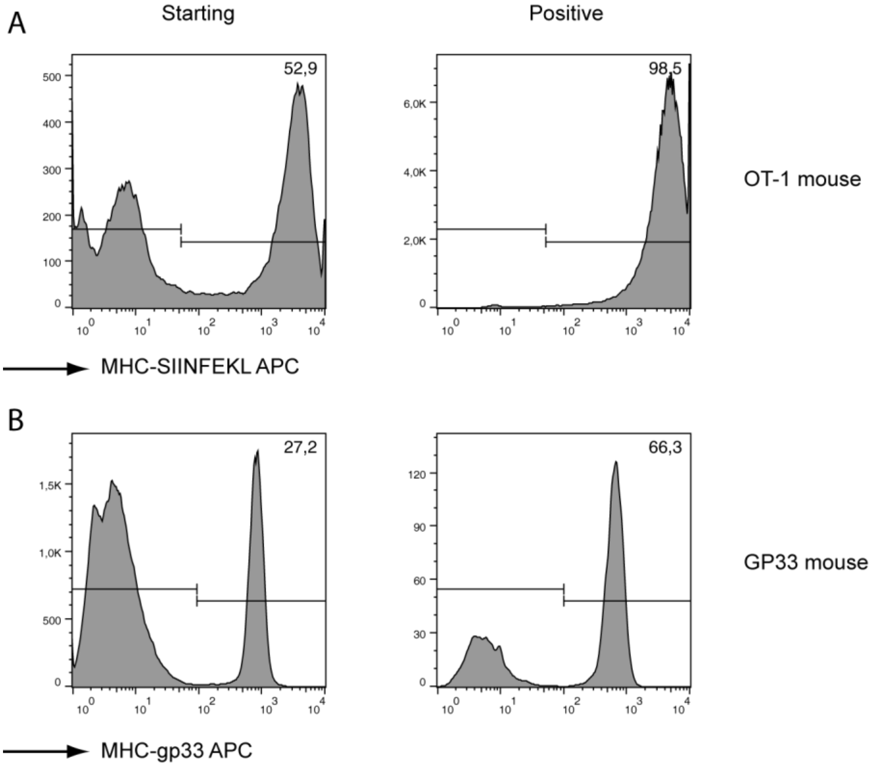

Enrichment of cell populations using affinity chromatography: A) Enrichment of SIINFEKL positive cells from mouse splenocytes of a OT-1 mouse; cells are gated on single, living events excluding debris and stained with MHC-SIINFEKL Streptamer. **B)** Enrichment of gp33 positive cells from mouse splenocytes of a GP33 mouse; cells are gated on single, living events excluding debris and stained with MHC-gp33 Streptamer
